# What is your diagnosis?

**DOI:** 10.4103/2589-0557.69010

**Published:** 2010

**Authors:** Khozema Saify, P. K. Saraswat, Dinesh Mishra, Pulak Jeswani

**Affiliations:** Department of Dermatology, Venereology and Leprology, G R Medical College, Gwalior, India

A 60-year-old uncircumcised male presented with asymptomatic growth on the glans penis of 7 years duration. There were no complaints or past history suggestive of sexually transmitted diseases (STDs). There was no history of trauma to penis, localized dermatoses, systemic diseases or any prolonged medications. There was no history of any significant illness in the partner. The condition was initially diagnosed clinically as a case of penile psoriasis and treated with potent topical corticosteroids. There was partial response to the treatment with some reduction in scaling, but the patch gradually increased in size and became elevated over the course of time. Over the past 1 year, the lesion started increasing in the size, developed verrucosity and thick mica-like scaling. On examination, there was hyperkeratotic, hypertrophic, verrucous plaque with thick scaling on the glans and rim of erythema. His S. VDRL and S. HIV tests were normal. Hematological, biochemical and radiological examination did not reveal any abnormality. Histopathological examination with hematoxylin and eosin staining showed irregular exo-endophytic hyperplasia of the epidermis with elongated downgrowths. There was prominent mitotic activity of the basal and suprabasal layers of the epidermis with mild atypia and pleomorphism of nuclei. The surface showed a marked thick, parakeratotic stratum corneum. A focally lichenoid lymphoplasmacytic infiltrate was present [Figures 1 and 2].

**Figure 1:**
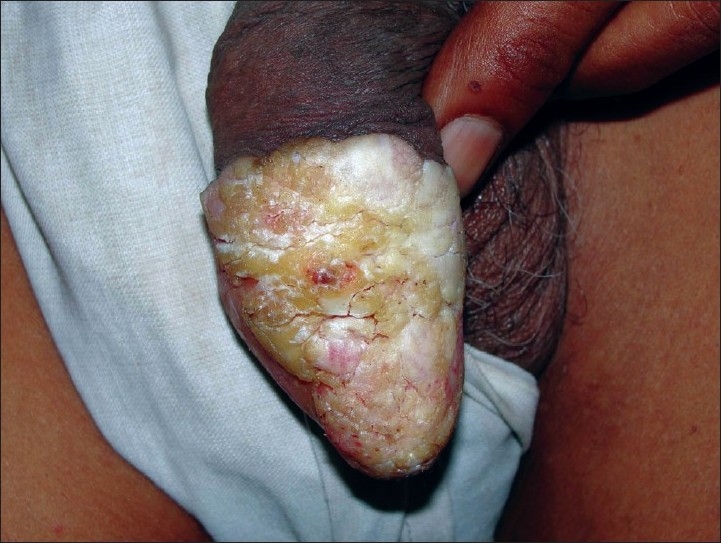
Hyperkeratotic plaques on the glans penis

**Figure 2:**
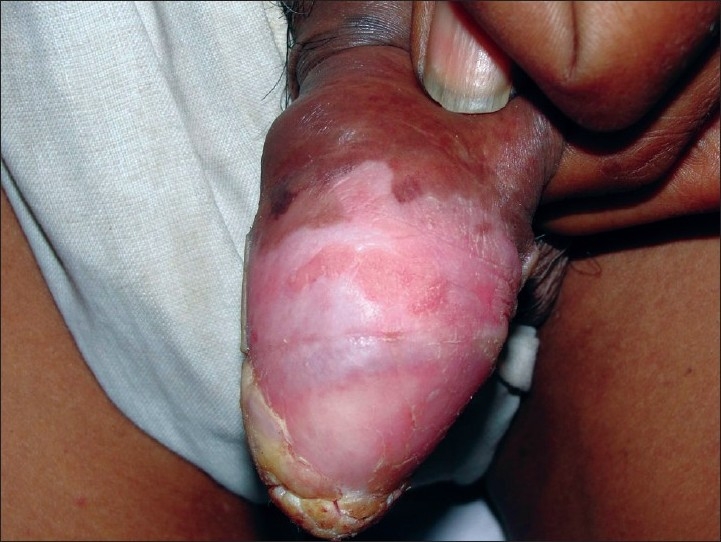
Erythema of the dorsal surface of the penis

## What is your diagnosis?

Pseudoepitheliomatous, keratotic and micaceous balanitis (PKMB)

## DISCUSSION

PKMB is a rare condition involving the skin of the glans penis that occurs in older uncircumcised men or most circumcised men late in life.[1] It presents as thick scaly micaceous patches (possibly a cutaneous horn) on the glans penis.[2] The etiology of this condition is unknown.[1] This condition was considered pseudomalignant, premalignant or as a low-grade squamous malignancy.[3] PKMB presents as a slowly enlarging hyperkeratotic plaque on the glans penis.[4] In the present case, the condition started as a hyperkeratotic plaque on the glans penis. Because of the rarity of the condition and lack of biopsy findings, the condition was provisionally diagnosed as a case of penile psoriasis, which could have been the obvious clinical possibility at that time. Later on, when the patient started developing complaints of foul smelling maceration, he was diagnosed as a case of monilial balanitis. A timely biopsy was needed to make a diagnosis, the lack of which caused the disease to linger on for a long period. Histological examination shows hyperkeratosis, parakeratosis, acanthosis, prolongation of rete ridges and mild lower epidermal dysplasia, with a nonspecific dermal inflammatory infiltrate of eosinophils and lymphocytes.[5] The spectrum of histologic findings may range from hypertrophic-hyperplastic penile dystrophy to verrucous carcinoma.[4] A role of human papilloma virus (HPV) has been suggested in the malignant transformation of benign condition into malignant disease.[6] However, in the case of Child *et al*., HPV DNA was not identifiable using broad-spectrum polymerase chain reaction in the lesion of verrucous carcinoma arising out of PKMB lesion. This condition was originally thought to be benign. Presently, it is considered to be of uncertain malignant potential and has been associated with progression to verrucous carcinoma and squamous cell carcinoma.[1,7] Sometimes, fibrosarcoma has been noted to develop.[8] Treatment depends on severity, and may range from topical treatment to surgical excision.[1] Topical treatment includes 5-FU, cryotherapy, electrocoagulation and shavebiopsy.[1,9,10] There have been reports of recurrence of the lesion after topical therapy.[9] Because evidence from published literature suggests that this lesion may have locally invasive or aggressive tendencies and that it should be considered to have low-grade or limited malignant potential, conservative surgical removal seems to be adequate therapy, which offers good prognosis.[11] In our case, because the lesion persisted for a long duration, and although biopsy did not suggest evidence of malignancy, a partial amputation of the penis was performed with removal of tumor and securing a clear tissue margin to prevent the development of carcinoma later in life. Sentinel lymph node biopsy was performed to rule out carcinomatous process.
